# Evaluation of the STANDARD M10 and Xpert *C. difficile* assays for the detection of toxigenic *Clostridioides difficile* in stools

**DOI:** 10.1128/spectrum.03470-25

**Published:** 2026-04-29

**Authors:** Y. Roukoz-Diab, M. Ehmig, S. Luce, C. Eckert, S. Timsit, J. Couturier, I. Mostaghat, F. Barbut

**Affiliations:** 1 National Reference Laboratory for Clostridioides difficile, AP-HP, Hôpital Saint-Antoine 37117, Paris, France; 2 Department of Clinical Bacteriology, AP-HP, Hôpital Saint-Antoine 37117, Paris, France; 3 Sorbonne Université, INSERM 1135, CIMI 27063, Paris, France; 4 Université Paris Cité, INSERM 1139, FPRM 555089, Paris, France; Johns Hopkins University, Baltimore, Maryland, USA

**Keywords:** *Clostridioides difficile *infection, diagnosis, NAAT, Ct (cycle threshold) values, toxigenic culture

## Abstract

This study aims to compare the sensitivity and specificity of two Nucleic Acid Amplification Tests (NAATs), the STANDARD M10 *Clostridioides difficile* (M10) and the Xpert *C. difficile* (Xpert) assays, for the detection of toxigenic *C. difficile* strains in stools. This prospective study included fresh diarrheal stools. Following routine testing by the *C. difficile* Quik Chek Complete enzyme immunoassay (EIA), M10 and Xpert were tested in parallel alongside toxigenic culture (gold standard) on selective chromogenic medium (ChromId *C. difficile*). The M10 and Xpert assays were performed by independent technicians. We also compared the results of the M10 assay from 10 native stools and fecal swab paired samples. A total of 541 stool samples were included. The toxigenic culture was positive for 60 stool specimens (11.0%), including 32 free toxin-positive stools. Both M10 and Xpert demonstrated comparable sensitivity (100%) and specificity (97.5%). The *tcdB* cycle threshold (Ct) values obtained with the M10 and Xpert assays were highly correlated (Pearson correlation coefficient: 0.909, *P* < 0.001). The median *tcdB* Ct values for toxin EIA-positive/NAAT-positive specimens were significantly lower than those for toxin EIA-negative/NAAT-positive specimens in both M10 (25.50 vs 32.39) and Xpert assays (23.70 vs 31.15) (*P* < 0.001). Receiver operating characteristic curve analysis assessing the ability of Ct values to predict the detection of free toxins exhibited an area under the curve of 0.875 for M10 and 0.862 for Xpert. The use of 400 µL of fecal swab transport medium yielded Ct values closer than 100 or 200 µL to those from native stools. The M10 *C. difficile* assay displays high sensitivity and specificity and is comparable to the Xpert assay.

**IMPORTANCE:**

Our study evaluated the performances of a new NAAT assay (STANDARD M10 *Clostridioides difficile* assay) for detecting toxigenic *C. difficile* in stool specimens and compared the results to the Xpert *C. difficile* assay, a widely used assay in clinical laboratories. This prospective study was conducted on a very large collection of 541 diarrheal fresh stool samples, and all *C. difficile* isolates were characterized by PCR-ribotyping. As of today, there are limited data regarding the performances of the STANDARD M10 *C. difficile* assay.

## INTRODUCTION

*Clostridioides difficile* is the main etiologic agent of antibiotic-associated diarrhea (AAD) and pseudomembranous colitis (PMC). It is the major agent of healthcare-associated diarrhea among hospitalized patients (1). Recently, *C. difficile* has also been recognized as an emerging pathogen in the community (2, 3). Clinical symptoms range from mild diarrhea to severe colitis and possibly to bowel perforation, septic shock, and death. Asymptomatic carriage of a toxigenic strain is usually observed in 3% of the general population, which may result in diagnostic issues (4). The predisposing risk factors for *C. difficile* infection (CDI) include advanced age, previous antimicrobial therapies, length and number of hospital stays, immunosuppressive treatments, and co-morbidities (diabetes, cardiovascular syndromes, renal failure) (1).

The landscape of CDI diagnosis has evolved over the last 15 years and still remains challenging. A rapid and accurate CDI diagnosis is crucial to guide the patient’s treatment and timely implement contact precautions. Standardization of CDI diagnosis is also essential to monitor CDI incidence over time and compare incidence across different healthcare facilities.

According to the European Society of Clinical Microbiology and Infectious Diseases (ESCMID) guidelines (5), the reference strategy for CDI diagnosis is based on a two-step algorithm starting with a screening method with a high sensitivity, followed, in case of a positive result, by a more specific method detecting free toxins in the stool. One option is to use a nucleic acid amplification test (NAAT) to detect toxin genes as a screening method. In case of a positive result, an enzyme immunoassay (EIA) detecting free toxins is recommended to confirm the diagnosis.

Many NAAT assays are now commercially available on the global market (6). Some tests are designed for laboratories with a low volume of activity or for point-of-care testing, while others are capable of high throughput. These tests usually detect the genes encoding toxins B (*tcdB*) and possibly additional genes, such as those encoding toxin A (*tcdA*) and/or the binary toxins (*cdtA* and *cdtB*). Genes for *C. difficile* toxins are often included in gastrointestinal panels (syndromic diagnosis), which enable the simultaneous detection of several bacterial, parasitic, and viral gastrointestinal pathogens in stool samples.

The STANDARD M10 *C. difficile* assay (M10, SD Biosensor, Suwon, Republic of Korea) is a new fully automated real-time PCR assay for *tcdB* gene detection from stool samples. As of the time of manuscript submission, there are limited data regarding the performances of this assay. The primary objective of this study was to compare the performance (sensitivity, specificity, predictive positive and negative values, expressed with their 95% confidence intervals) of the M10 *C. difficile* assay and compare the results to the Xpert *C. difficile* assay (Xpert, Cepheid, Sunnyvale, CA, USA) for detecting toxigenic *C. difficile* in stool specimens. The gold standard used for this comparison was the toxigenic culture. The secondary objective was to compare the M10 assay results on native stools to stools sampled with a fecal swab (Copan, Brescia, Italy) in order to assess the impact of the Cary-Blair medium used for stool preservation.

## MATERIALS AND METHODS

### Stool selection

This prospective study was conducted at the Clinical Bacteriology Laboratory of Saint Antoine University Hospital (Paris, France) from 15 October to 3 December 2024 and from 15 March to 20 April 2025. Fresh unformed stools (defined as stools taking the shape of the container) submitted for *C. difficile* testing were included in the study. Formed stools, stools from infants less than 2 years old, and repetitive stool samples within a period of 7 days were excluded from the study. All stool samples were analyzed using *C. diff* Quik Chek Complete (Techlab, Blacksburg, VA, USA) within 48 h of receipt as part of the hospital standard two-step algorithm diagnostic routine. All study samples were subsequently tested on the same day by culture and by M10 and Xpert *C. difficile* assays by three independent technicians blinded to prior test results. An aliquot of each original stool sample was stored at −80°C for further testing.

### GDH and free toxins detection assay

Each stool sample was tested for the presence of glutamate dehydrogenase (GDH) and free toxins (A and B) with *C. diff* Quik Chek Complete enzyme-immunoassay (EIA) according to the manufacturer’s instructions.

### Nucleic acid amplification test (NAAT)

Both M10 and Xpert *C. difficile* assays detect *tcdB* gene only. They were performed according to the manufacturer’s instructions by two independent technicians. For the M10 assay, a swab was dipped into a thoroughly mixed stool sample, transferred into the pretreatment solution, and mixed using a vortex for 10 s. The hands-on time was minimal (<10 min). The sample was loaded into the cartridge, which was placed into the machine module for nucleic acid extraction and amplification (40 cycles). The results were automatically interpreted and available in less than 50 min. The amplification curves were provided. For the Xpert *C. difficile* assay, samples with a Ct (cycle threshold) higher than 38 were considered as negative per the manufacturer’s instructions. If any specimens exhibited an invalid result with M10 or Xpert, then they were immediately retested using the original specimen.

### Toxigenic and enriched culture

Stool samples were cultured on selective ChromID *C. difficile* agar (biomérieux, Marcy-l’Etoile, France) and incubated for 48 h at 37°C in an anaerobic atmosphere. Suspicious colonies (black or white colonies with a typical *C. difficile* aspect) were confirmed using matrix-assisted laser desorption/ionization-time-of-flight (MALDI-TOF) mass spectrometry (Bruker Daltonics, Germany). The toxigenic status of confirmed isolates was determined using a multiplex PCR detecting virulence factors as described below.

### Molecular characterization of *C. difficile* isolates

DNA extraction was performed from colonies grown on Brucella agar (bioMérieux, Marcy-l’Etoile, France) using the InstaGene Matrix Kit (Bio-Rad, Marnes-la-Coquette, France). Strains were characterized by a previously described multiplex PCR assay detecting *tpi*, *tcdA*, *tcdB*, *tcdC*, *cdtA*, and *cdtB* genes coding for the triose phosphate isomerase, toxin A, toxin B, TcdC, and the two components of the binary toxin, respectively (2).

Isolates were characterized by capillary gel electrophoresis-based PCR ribotyping on an ABI 3500 sequencer (Applied Biosystems, Foster City, CA, USA) using primers described by Bidet et al. (7). After DNA amplification, each PCR product was diluted at 1/200. One microliter of this dilution was mixed with 10.5 µL formamide and 0.5 µL GeneScan LIZ1200 (Applied Biosystems, Foster City, USA). Banding patterns were analyzed with the GeneMapper software (Thermo Fisher Scientific, Villebon-sur-Yvette, France). PCR ribotypes (RT) were assigned using the Webribo database (https://webribo.ages.at/).

### Discordant result analysis

If the M10 and Xpert assays results were discordant, then both assays were performed again, and the second result was considered as the final result. For samples with a negative culture and a positive NAAT or GDH-EIA result, an enriched culture was performed from the stool aliquot stored at −80°C. Briefly, a pea-size sample of stool was inoculated in a brain-heart infusion broth (bioMérieux) containing cefoxitin (10 μg/mL), cycloserine (250 μg/mL), and 0.1% taurocholate and incubated at 37°C for 5 days in an anaerobic atmosphere. Then, the broth was subcultured on ChromID *C. difficile* plates for 48 hours at 37°C.

### Evaluation of the M10 assay from stools collected with Copan fecal swab

We compared the M10 assay Ct values from fresh stools to stools diluted in a modified Cary-Blair medium (FecalSwab, Copan). Ten fresh stools, including nine positive and one negative with M10, were randomly selected. In parallel, a flocked fecal swab was dipped into these stools, then discharged into 2 mL of the Cary-Blair medium, which stayed at room temperature for 2 h. Then, 100, 200, and 400 µL of the stool suspension were placed into the pretreatment solution, and the M10 assay was performed for each volume of sample, as previously described.

### Statistical analysis

The results of both NAATs were first compared to the result of the toxigenic culture considered as the gold standard. The performance of the M10 and Xpert assays was evaluated in terms of sensitivity, specificity, positive predictive value (PPV), and negative predictive value (NPV) (https://www.medcalc.org/). After resolving the discrepant results by enriched culture, the performance of NAATs was recalculated.

The Mann-Whitney *U* test was used to compare the median *tcdB* cycle threshold (Ct) values between toxin EIA-positive/NAAT-positive and toxin EIA-negative/NAAT-positive groups. Receiver operating characteristic (ROC) curves were used to assess the ability of Ct values to predict toxin EIA results, with optimal cut-off value determined using Youden’s index.

The correlation between Ct values obtained from both M10 and Xpert assays was assessed using simple linear regression, and the Pearson correlation coefficient (r) was calculated. A *P* value below 0.05 was considered statistically significant. All statistical analyses were performed using Prism 8.0.1 GraphPad software.

## RESULTS

A total of 541 consecutive diarrheal stool specimens were included in the study. Stool consistency was described as liquid (*n* = 220, 40.6%), loose (*n* = 313, 57.8%), or hemorrhagic (*n* = 8, 1.4%). Out of these samples, 15 (2.7%) gave an initial invalid result with Xpert and none (0%) with M10 assays. Seventy-eight stool samples (14.4%) turned out to be positive in culture (*n* = 72)/enriched culture (*n* = 6). Among the corresponding strains, 60 (11.1%) were toxigenic (positive for both *tcdA* and *tcdB* genes), and 18 (3.3%) were considered as non-toxigenic. Among the 60 stools positive with a toxigenic strain, free toxins (*C. diff* Quik Chek Complete immunoassay) were detected in only 32 (53.3%).

Toxigenic strains belonged to RT 014 (*n* = 7), 106 (*n* = 7), 126 (*n* = 4), 002 (*n* = 4), 020 (*n* = 3), 404 (*n* = 3), 001 (*n* = 3), 078 (*n* = 2), 015 (*n* = 2), 236 (*n* = 2), 076 (*n* = 2), 651 (*n* = 2), and others (*n* = 19). Seven strains (RT 78, RT 126, and others) were also found positive for the binary toxin *cdtB* and *cdtA* genes.

The performance of both M10 and Xpert for detecting a toxigenic *C. difficile* strain from stools is summarized in Tables 1 and 2. Compared to toxigenic culture, both NAATs displayed a very high sensitivity and specificity (>97%). After resolving discrepancies by enriched culture, the sensitivity and specificity of M10 and Xpert still remained very high (>98%) (Tables 1 and 2). The six M10 false positive results displayed a Ct of 35.51, 35.69, 31.8, 34.95, 34.47, and 35.31, whereas the seven Xpert false positive results displayed a Ct of 36.3, 35.5, 35.1, 35.6, 34.7, 36.4, and 34.0 (Table 3). Overall, five stool samples gave false positive results by both NAATs. Interestingly, GDH testing, which is often used as the screening test in a two-step algorithm, missed six toxigenic culture-positive samples, leading to a sensitivity for detecting toxigenic strains of 92.3% [79.4–96.2]. These six false negative GDH results corresponded to stool samples with high Ct with M10 (34.10, 35.42, 34.79, 34.76, 32.30, and 35.49) and Xpert (35.20, 36.00, 34.50, 31.60, 30.10, and 38.60).

**TABLE 1:** Clinical performances (sensitivity, specificity, positive and negative predictive values, accuracy) of M10 and Xpert for detecting C. *difficile* toxigenic strains from stools vs toxigenic culture

	Sensitivity (CI 95%)	Specificity (CI 95%)	NPV (CI 95%)	PPV (CI 95%)	Accuracy (CI 95%)
Xpert assay	100 (93.4–100.0)	97.5 (95.7–98.7)	100 (99.2–100.0)	81.8 (72.0–88.7)	97.8 (96.2–98.8)
M10 assay	100 (93.4–100.0)	97.5 (95.7–98.7)	100 (99.2–100)	81.8 (72.0–88.7)	97.8 (96.2–98.8)

**TABLE 2:** Clinical performances (sensitivity, specificity, positive and negative predictive values, accuracy) of M10 and Xpert for detecting *C. difficile* toxigenic strains from stools after resolving discordant results by enriched toxigenic culture^*a*^

	Sensitivity (CI 95%)	Specificity (CI 95%)	NPV (CI 95%)	PPV (CI 95%)	Accuracy (CI 95%)
Xpert assay	98.3 (91.0–99.9)	98.5 (97.0–99.4)	99.7 (98.5–99.9)	89.3 (80.1–94.6)	98.5 (97.1–99.3)
M10 assay	100 (94.0–100)	98.7 (97.3–99.5)	100 (99.2–100)	90.9 (81.8–95.6)	98.8 (97.6–99.5)

^*a*^ NPV, negative predictive value; PPV, positive predictive value; and CI, confidence interval.

**TABLE 3:** Description of false positive results obtained with M10 and Xpert as compared to culture/toxigenic culture^*a*^

	STANDARD M10			Xpert Cepheid			Quik Chek Complete		Culture	Final interpretation	Strain characterization		
Specimen #	Ct IC	Ct *tcdB*	Interpretation	Ct IC	Ct *tcdB*	Interpretation	GDH	Free toxins			Ribotype	*tcdB*	*tcdA*
EB24-456	27.66	35.51	Pos	30.30	36.30	Pos	+	−	+	Pos	039	−	−
EB24-241	26.58	35.69	Pos	31.50	35.50	Pos	−	−	EC−	Neg	NA	NA	NA
EB24-301	27.70	31.80	Pos	29.60	38.70	Neg	+	−	EC−	Neg	NA	NA	NA
EB24-486	26.36	34.95	Pos	31.20	35.60	Pos	+	−	EC−	Neg	NA	NA	NA
EB24-519	27.43	34.47	Pos	31.20	34.70	Pos	+	−	EC−	Neg	NA	NA	NA
EB24-529	27.37	35.31	Pos	30.50	36.40	Pos	−	−	EC−	Neg	NA	NA	NA
EB24-035	26.55	N/A	Neg	29.60	35.10	Pos	+	−	+	Pos	FR377	−	−
EB24-125	27.62	N/A	Neg	30.60	34.00	Pos	+	−	EC−	Neg	NA	NA	NA

^*a*^ IC, internal control; EC, enriched culture; Pos, positive; Neg, negative; and NA, not applicable. Cycle threshold cut-off values were set at 38 and 40 cycles for Xpert and STANDARD M10, respectively.

**Fig 1:**
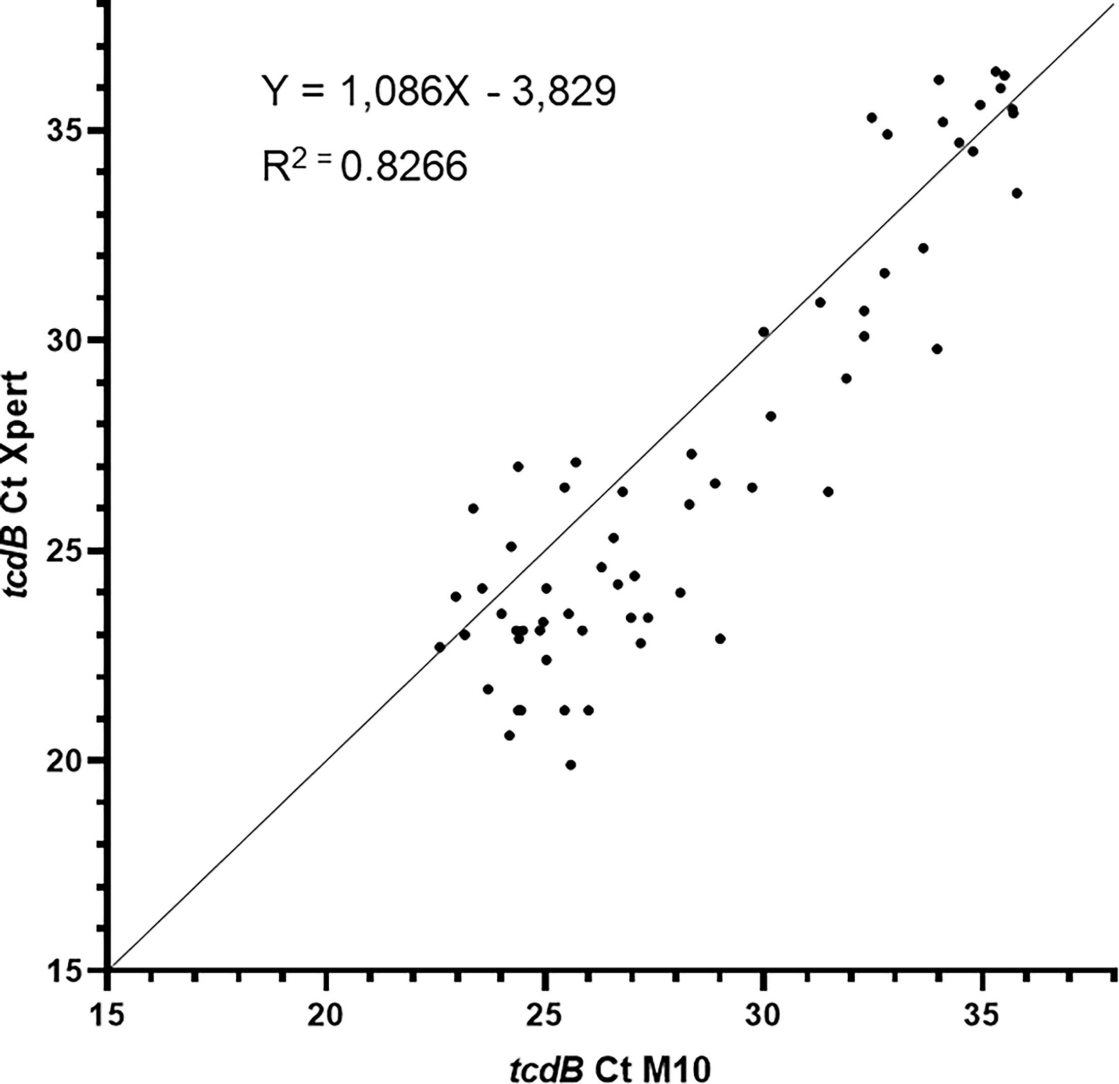
Correlation between M10 and Xpert Ct values for the *tcdB* gene detection.

The Ct values obtained from the M10 and the Xpert assays were highly correlated, with a Pearson correlation coefficient of 0.909 (95% confidence interval: 0.854–0.944, *P* < 0.0001) (Fig. 1).

**Fig 2:**
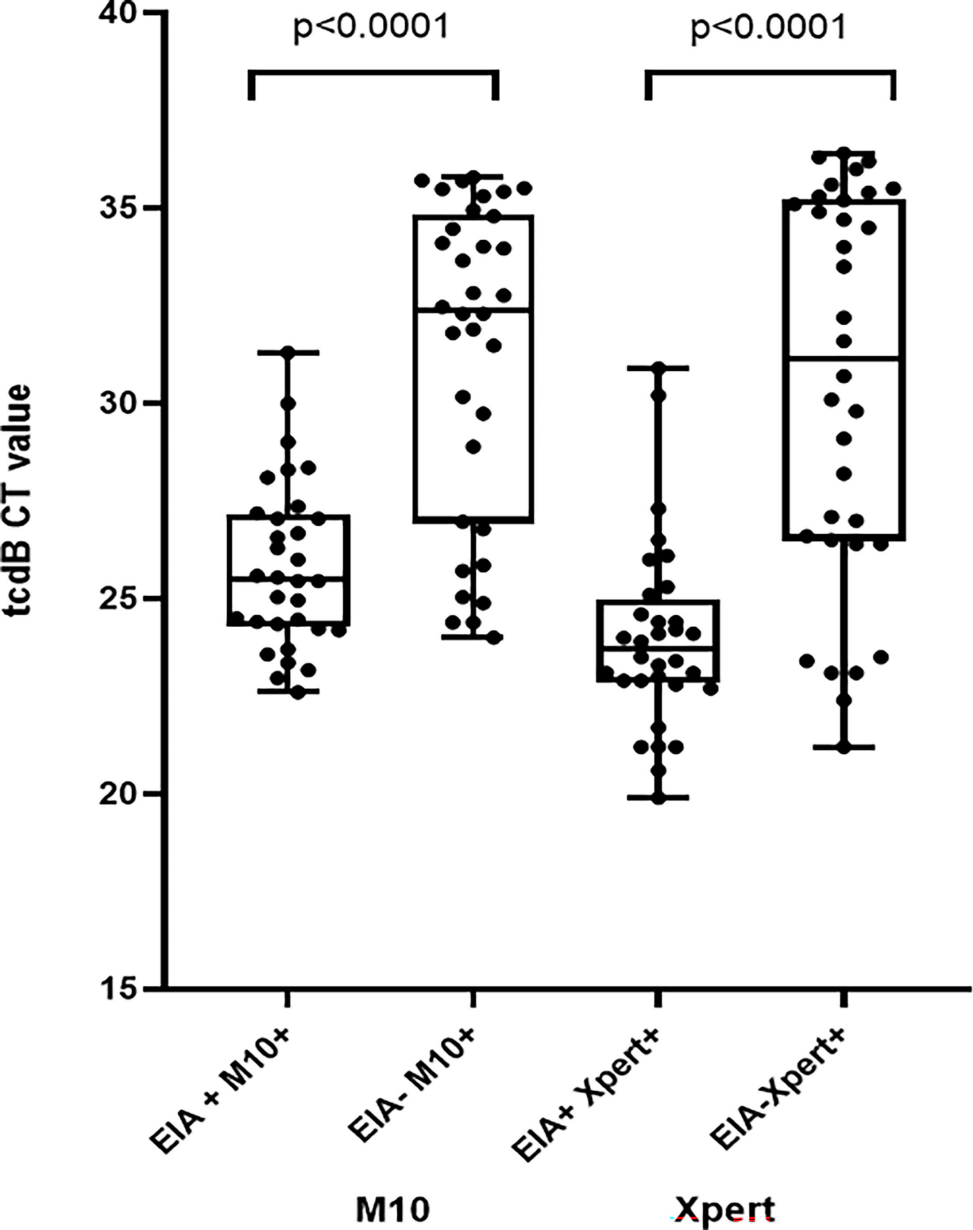
Comparison of M10 and Xpert assay Ct values in patients who were toxin-EIA positive in stools to those who were toxin-EIA negative.

Ct values between the toxin EIA-positive/NAAT-positive group and EIA-negative/NAAT-positive group are compared in Fig. 2. The median Ct values of each NAAT were significantly lower in patients who were toxin-EIA positive in stools compared to those who were toxin-EIA negative (25.50 versus 32.39 for the M10, *P* < 0.0001, and 23.70 versus 31.15 for the Xpert, *P* < 0.0001). Nevertheless, the Ct values between EIA-positive and EIA-negative populations exhibited a significant overlap.

**Fig 3:**
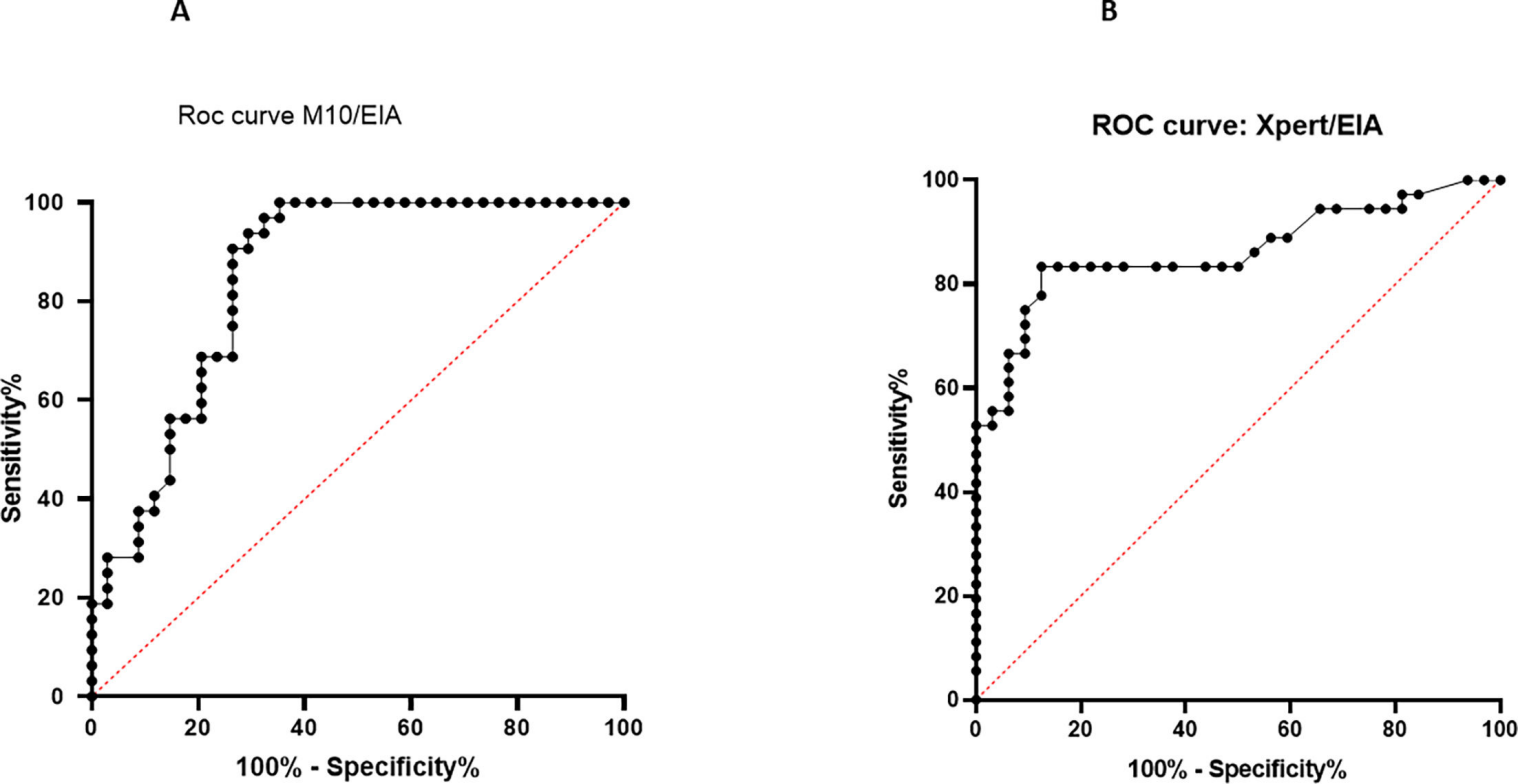
Receiver operating curve (ROC) of M10 (3A) and Xpert (3B) assessing the ability of Ct values to predict the detection of free toxins in stools.

The ROC curve analysis, which assessed the ability of Ct values to predict toxin EIA results, revealed an area under the curve (AUC) of 0.875 (0.750–1.00) and 0.862 (0.772–0.953) for M10 and Xpert assays, respectively (Fig. 3). The optimal cut-off values for the M10 and Xpert assays were 31.4 and 26.2, respectively, using the Youden’s index. Nine positive stool specimens and one negative (control) were simultaneously tested with M10 on native stools and on stools sampled with a fecal swab. Different volumes (100, 200, and 400 µL) of the fecal swab collection broth were used to perform the M10 assay. Native stools and fecal swab Ct values were compared. The results are presented in Table 4. Only one specimen positive on native stools with a very high Ct (35.79) turned out to be negative when using 100 µL volume of fecal swab broth. The use of a volume of 400 µL gave Ct values (mean 28.25) close to that of native stools (mean 28.18)*.*

**TABLE 4:** Comparison of M10 assay Ct values from native stools and stools diluted in the Cary-Blair medium (FecalSwab, Copan)^*a*^

Specimen #	Native stool			Fecal swab					
	Ct IC	Ct *tcdB*	Interpretation	Ct IC 100 µL	Ct 100 µL	Ct IC 200 µL	Ct 200 µL	Ct IC 400 µL	Ct 400 µL
EB24-036	26.08	23.57	Pos	26.94	26.90	26.59	26.46	25.82	25.20
EB24-034	26.98	Neg	Neg	26.88	Neg	27.15	Neg	26.72	Neg
EB24-073	27.95	34.10	Pos	26.39	35.15	26.62	34.59	26.84	34.60
EB24-074	27.12	26.67	Pos	26.75	26.71	26.28	25.46	26.48	24.56
EB24-090	26.88	24.23	Pos	26.81	26.76	26.48	25.35	26.43	24.82
EB24-116	26.90	26.00	Pos	26.50	24.91	26.71	23.90	25.55	23.02
EB24-123	27.10	35.79	Pos	27.25	Neg	27.27	36.86	27.24	35.90
EB24-124	26.75	25.45	Pos	26.78	27.88	27.00	26.91	26.68	26.45
EB24-126	26.78	24.19	Pos	26.68	26.14	26.83	25.33	26.60	24.66
EB24-127	27.03	33.65	Pos	26.88	34.79	26.73	34.42	27.07	35.03

^*a*^ IC, internal control; Ct, cycle threshold; Pos, positive; and Neg, negative.

## DISCUSSION

This study aimed to compare the performance of the M10 *C. difficile* assay, a new multiplex real-time PCR assay, with that of the Xpert *C. difficile* assay, a widely used NAAT in clinical laboratories, for the detection of toxigenic C. *difficile* in stools. This prospective study was conducted on a very large collection of 541 diarrheal fresh stool samples, and all *C. difficile* isolates were characterized by PCR-ribotyping. Results indicated that both M10 and Xpert *C. difficile* assays display an outstanding sensitivity (100%) compared to toxigenic culture. This finding is consistent with a previous study showing a sensitivity of 100% for both methods using the same reference method (toxigenic culture) (8, 9). The sensitivity of both NAATs was higher than that of GDH detection, as shown by other studies and strengthens the fact that NAAT assays perform better as a screening method in a two-step diagnostic algorithm (5, 10, 11). The specificities of M10 and Xpert *C. difficile* assays were identical (97.5%). After resolving the discrepant results by enriched culture, the sensitivity of Xpert slightly decreased (98.5%), while the specificity increased for both NAAT assays (98.5 and 98.7% for Xpert and M10, respectively). Among the remaining eight false positive results, five were positive by both NAATs; one was positive with STANDARD M10 only; and two were positive with Xpert *C. difficile* only. Interestingly, among these eight false positive results, six were positive for GDH detection: of these, four turned out to be negative by the enriched culture, and two were positive with a non-toxigenic strain (RT 039 and FR 377). The Ct values of these false positive NAAT results were all above 30, suggesting a very low burden of *C. difficile*, very likely below the detection threshold of enriched culture. Another hypothesis to explain the discrepancies between NAAT-positive and enriched culture-negative results would be the presence of residual *C. difficile* DNA following an antibiotic treatment active on *C. difficile* vegetative cells. This hypothesis could not be checked because of the lack of clinical information on CDI patients.

The role of NAAT in the diagnosis of CDI is still a matter of debate. Indeed, a positive NAAT result is unable to differentiate a true infection from a simple colonization by a toxigenic strain. Several studies have shown that detection of free toxins in stool samples is better correlated with the severity of disease (12, 13). The use of Ct values obtained from NAAT assays has been proposed to predict the presence of free toxins. As a result, the ESCMID has proposed a diagnosis of CDI based on a clinical presentation compatible with CDI and a positive NAAT preferably with a low Ct value (4, 6, 7). Our ROC curve analysis revealed AUC values of 0.875 and 0.862 for the M10 and the Xpert assays, respectively, supporting that Ct values could be helpful to predict toxin EIA results, as suggested by a previous study using the same NAAT assays (8, 9). The optimal Ct cut-off values obtained in our study (31.4 and 26.2 for the M10 and Xpert assays, respectively) were close to those found by Lee et al*.* (30.7 and 27.4, respectively) who used the same assay (Quik Chek Complete assay) for free toxin detection (8). However, the Ct value to predict free toxins in stools and distinguish infection from colonization must be used carefully for several reasons. First, it is impossible to determine a universal Ct value due to different amplification efficiencies of NAAT assays. Second, the determination of the Ct value threshold is highly dependent upon the EIA used. Third, it is well known that every *C. difficile* ribotype does not produce the same amount of toxins. Fourth, the release of toxins is highly dependent on environmental conditions in the gut that up- or downregulate genetic elements within the PaLoc. Therefore, a laboratory wishing to use Ct values to predict free toxins in stools will need to validate its use.

Finally, the secondary objective of the study was to compare the performance of fecal swabs with the liquid transport medium to native stools for toxigenic *C. difficile* detection. Since the increasing use of microbiology automation, many routine laboratories now receive stool samples in fecal transport medium rather than native stool samples. In a previous study, Jazmati et al. (14) have shown that the use of fecal swabs may decrease the performance of *C. difficile* detection by culture, PCR, or GDH probably due to a dilutional effect of the fecal sample in the liquid medium (14). To assess the impact of transport medium, we compared the Ct values obtained with the M10 assay between fresh stools and stools diluted in the Cary-Blair medium. Our results indicated that the Ct values obtained with fecal swabs were slightly higher compared with native stools, suggesting that the impact of transport medium was minimal. We recommend using 400 µL rather than 100 µL of Cary Blair medium to perform M10 to overcome the false negative result that may happen for native stools with a very high Ct.

Our study presents several limitations. First, the study involved a single laboratory and may not be generalizable to other healthcare settings with different ribotype distributions and CDI epidemiologies. Second, the evaluation compared the performance of NAAT to toxigenic culture and not to the clinical CDI diagnosis. Lastly, enriched toxigenic culture was only performed in case of discrepant results and not for all stool specimens. Therefore, it is possible that we underestimated the prevalence of positive culture and consequently overestimated the sensitivity of NAAT assays.

In conclusion, our study demonstrated that M10 *C. difficile* assay displays high sensitivity and specificity for detecting toxigenic strains and performed comparably to the Xpert assay, which is widely used in clinical laboratories.
